# A comparison of group art therapy in decreasing the depression level of leukemia pediatric patients

**DOI:** 10.1192/j.eurpsy.2021.1153

**Published:** 2021-08-13

**Authors:** A. Naveed, S. Masood

**Affiliations:** 1 -, private, karachi, Pakistan; 2 Department Of Psychology, University of Karachi, Karachi, Pakistan

**Keywords:** group art therapy, Depression, pediatric leukemia

## Abstract

**Introduction:**

Cancer and its treatment often impose physical and psychological consequences. Children with cancer are not only at a risk of adverse events resulting from medical procedures but also severe effects on their social and mental health as a result of its treatment. Depression being one of the most common psychiatric disorders associated with cancer in children and adolescents. The current study aims to provide evidence of an easy and inexpensive intervention that can be used in oncology patients in a low resource setting to help maintain mental health stability in pedriatric patients..

**Objectives:**

To examine the effectiveness of therapeutic group art therapy, in reducing depression scores in children with cancer.

**Methods:**

The study is quasi experimental, through convinient sampling data of fifteen children in initial stage of thier treatment were selected. They were divided into two groups experimental and controlled through random allocation. Three sessions of forty minutes of group art therapy was given in experimental group, in control normal treatment was provided excluding group art therapy. Childhood Depression Scale (CDI) was used in both group as pre and post test to determine the depression level.

**Results:**

The results shows a positive change in the level of depression in experimental group as compared to controlled group.

**Conclusions:**

Group art therapy is an effective and inexpensive way of reducing depression level of paediatric leukemia patients that can be used by oncology healthcare centres worldwide with trained mental healthcare professionals in order to maintain positive treatment results of cancer.

